# Prevalence of Overhydration in Patients on Maintenance Haemodialysis As Determined by Body Composition Monitor and Effects of Attaining Target Dry Weight

**DOI:** 10.7759/cureus.29509

**Published:** 2022-09-23

**Authors:** Nidhish Chandra Mathilakath, Jayachandran Selvaraj, Sreejith Parameswaran, Stalin Viswanathan, Vivekanandan Pillai, Harichandrakumar KT

**Affiliations:** 1 General Medicine, Jawaharlal Institute of Postgraduate Medical Education and Research, Puducherry, IND; 2 Nephrology, Jawaharlal Institute of Postgraduate Medical Education and Research, Puducherry, IND; 3 Biostatistics, Jawaharlal Institute of Postgraduate Medical Education and Research, Puducherry, IND

**Keywords:** target dry weight, bioimpedance, overhydration, maintenance haemodialysis, body composition monitor

## Abstract

Introduction: Fluid overload in chronic kidney disease (CKD) is an independent risk factor for all-cause mortality. The volume of ultrafiltrate removed during haemodialysis is usually assessed clinically. Assessment of overhydration by body composition monitor (BCM) using bioimpedance spectroscopy is an objective method. This study was conducted to identify the prevalence of overhydration in CKD patients on maintenance haemodialysis and thereby assess the effects of BCM targeted dry weight attainment.

Methods: All patients included in the study were assessed for one month before enrolment for blood pressure, intradialytic events during each dialysis and BP medications. Overhydration was defined as the ratio of overhydration to extracellular water (OH/ECW) > 1.1. Overhydrated patients were brought to BCM targeted dry weight by increasing ultrafiltrate to 500mL/week more than their routine intradialytic weight gain. The effect of attaining BCM target dry weight on blood pressure and intradialytic events were analysed.

Results: Out of 110 patients, overhydration was seen in 30 (27.2%); only 20 had clinically evident overhydration. Body composition monitor guided dry weight was achieved in 28 of the 30 patients after a mean duration of 20 weeks. After achieving the target dry weight, there was a significant reduction in intradialytic hypertension events (2.37 vs 1.82 events per session, p-value 0.01). Surprisingly, there was a reduction in episodes of intradialytic hypotension as well, though this did not reach statistical significance. There was a clinically significant reduction in mean systolic and diastolic blood pressures (mean of 5.7mmHg and 2.8mmHg, respectively).

Conclusion: The study underlines the importance of BCM-based hydration status assessment and target dry weight attainment in better control of intradialytic events and blood pressure in patients on maintenance haemodialysis.

## Introduction

Achieving euvolemic status in patients on maintenance haemodialysis is extremely important. Inadequate fluid removal during dialysis leads to volume overload and chronic hypertension; excessive fluid removal leads to intradialytic hypotension. Both, increase mortality in patients with chronic kidney disease [1,2]. The dry weight is the weight at which the patient is close to euvolemia. This is the weight that needs to be achieved at the end of the dialysis session. This has also been defined as the lowest tolerated post-dialysis weight [3]. Several clinical clues are used to identify dry weight such as the absence of oedema and achievement of normal blood pressure without the use of antihypertensive drugs. However, they all have significant limitations. Identifying the dry weight of an individual patient remains a challenge for the clinician. Atrial natriuretic peptide (ANP) and cyclic guanosine monophosphate (cGMP) were considered biomarkers of volume overload [4,5]. Several objective methods have become available. Inferior vena cava diameter [6], bioimpedance spectroscopy (BIS), relative plasma volume (RPW) monitoring, and detection of lung water using chest ultrasound are some of the promising methods [7].

BIS is a simple, inexpensive, and non-invasive method [7-10]. It is rapid, takes about two minutes and can be used for repeat observations. It has been validated against gold standard techniques such as radionuclide dilution techniques for extracellular fluid (ECF), intracellular fluid (ICF) and total body water (TBW) volume determinations and DEXA scan for estimation of adipose tissue mass and lean tissue mass [7].

There is very limited data on the usage of this technique in the South Indian population. This study was undertaken to identify overhydration in patients on maintenance haemodialysis using BIS and then to bring the overhydrated patients to their body composition monitor (BCM) targeted dry weight. By doing so, we aimed at finding the effect of the bioimpedance technique on the frequency of intradialytic complications and blood pressure control.

## Materials and methods

This was a prospective observational study done at a tertiary care health centre in South India. The study was approved by the Institutional Ethics Committee (Human Studies), Jawaharlal Institute of Postgraduate Medical Education and Research (JIPMER), Puducherry. Subsequent to the approval, adult patients who had been on maintenance haemodialysis for a period of at least three months were enrolled after informed consent. Exclusion criteria were post-renal transplantation status, active malignancy, presence of coronary artery stent or cardiac devices such as implanted cardioverter defibrillators or cardiac pacemakers, presence of metal prostheses, artificial limbs, or extremity amputation, liver failure and pregnancy or lactation.

At enrolment baseline data were collected. After enrolment, during a one-month run-in period, blood pressure (BP) changes during dialysis were noted. Systolic blood pressure (SBP) and diastolic blood pressure (DBP) were checked during each dialysis session (every 30 minutes during dialysis, and immediately after dialysis), using automated BP apparatus (Fresenius Medical Care, Germany) in the non-fistula arm. Intradialytic hypotension and hypertension were noted. Intradialytic hypotension was defined as one or more of (1) SBP fall of ≥ 20mm Hg, (2) mean arterial pressure (MAP) fall of ≥ 10mmHg or (3) any fall in BP requires intervention (fluid bolus/reducing ultrafiltration/termination of haemodialysis) [11]. Intradialytic hypertension was defined as one or more of (1) SBP rise of ≥ 10mm Hg, (2) MAP rise of ≥15mmHg or more, or (3) any increase in BP that is resistant to fluid removal [12]. At the end of one month period, the study subjects underwent BCM assessment using BIS.

A BCM is a portable multifrequency machine (Fresenius medical care) that determines the hydration status of patients. The machine uses electrodes placed in the non-fistula limb (one electrode above and below the wrist and one electrode above and below the ankle separated by at least 3cm). The BCM device determines whole body impedance at 50 frequencies with very high precision. The frequencies used range from 5 kHz to at least 1,000 MHz Since a range of frequencies is used it is called BIS. From the impedance data, the ECW and TBW volumes are determined. Subsequently, using a physiological body composition model that is based on tissue properties, ECF excess or deficiency is calculated. This is called fluid overload (FO) and is expressed in litres. Normal hydration is defined as −1.1 to 1.1 (according to the 10th and 90th percentile of measurements in 1,242 healthy subjects) with values below and above this value defined as under and overhydration respectively [7].

The entire study group was divided into overhydrated and non-overhydrated groups. The overhydrated patients were brought to their BCM target dry weight by slowly increasing the ultrafiltrate at the rate of 500mL/week more than their routine intradialytic weight gain. Patients would be on their regular diet for CKD and reduced water intake plans. Once dry weight, as estimated by the BCM had been attained, they were followed up for one month during which blood pressure, intra-dialytic hypotension and hypertension and the number and dose of antihypertensive drugs were closely monitored.

Considering an expected prevalence of overhydration using a BCM to be 60%, with 15% relative precision and 5% level of significance the sample size was calculated as 114. Continuous variables were reported with mean or median and interquartile range (IQR). For categorical variables, the frequency with the percentage was presented. Comparisons of baseline characteristics were carried out using one-way ANOVA, analysis of homogeneity between groups or chi-squared tests for categorical variables. Post dry weight attainment variables were compared with baseline variables using paired t-test. Results were considered significant when p < 0.05.

## Results

The study was conducted from July 2018 to July 2020. We enrolled 110 patients undergoing maintenance haemodialysis. As per the BCM measurement done at the end of the first month, 30 of the 110 patients were found to be overhydrated. The overhydration index ranged from 1.2 to 6.4.

Table 1 shows the difference in baseline characteristics between the overhydrated and non-overhydrated groups. In comparing the two groups, there was a significant difference in intradialytic hypertension events between two groups. Overhydrated group was on dialysis for a longer duration when compared to the non-overhydrated group. There was no significant difference in weight, BCM-adjusted dry weight, or systolic or DBP. The mean duration required for attaining dry weight was 20 weeks. Of the 30 patients who were overhydrated, two patients were later excluded, one underwent a renal transplant and the other did not attain dry weight. Overhydrated patients were followed up and after attainment of dry weight, the effect on different variables was noted. 

**Table 1:** Baseline characteristics in overhydrated and non-overhydrated groups SD - Standard deviation, HD - Haemodialysis, BMI - Body mass index, BCM - Body composition monitor.

S. No	Variables	Expression	Overhydrated group, N=30	Non-overhydrated group, N=80	P-value
1.	Age (years)	Mean (SD)	43.9(13.9)	40(13.5)	0.19
2.	Duration of HD (years)	Mean (SD)	2(1)	1(2)	0.004
3.	Weight in kg	Mean (SD)	54.9(9.2)	55.1(11.5)	0.917
4.	Height in cm	Mean (SD)	161.3(7.8)	1160.1(10.1)	0.542
5.	BMI	Mean (SD)	20.9(3.1)	21.4(4.2)	0.574
6.	BCM adjusted dry weight	Mean (SD)	52.1(8.8)	55.7(11.5)	0.105
7.	Systolic blood pressure (mmHg)	Mean (SD)	150.5(15.7)	148.3(19.5)	0.559
8.	Diastolic blood pressure (mm of Hg)	Mean (SD)	81.6(12)	81.5(11.3)	0.967
9.	Number of anti-Hypertensive medications	Mean (SD)	2.4(1.2)	3.3(8.7)	0.195
10.	Urine output (ml)	Mean (SD)	294(353)	280(443)	0.866
11.	Intradialytic hypertension (events/session)	Mean (SD)	4.4(4.5)	1.6(2.2)	<0.001
12.	Intradialytic hypotension (events/session)	Mean (SD)	1.2(2.1)	0.7(1.3)	0.217

As shown in Table 2, there was a drop in mean SBP and DBP of 5.7mmHg and 2.83mmHg after attaining BCM targeted dry weight, however, the difference in pressures was not statistically significant. There was no significant difference in the number of BP drugs before and after attaining dry weight in the overhydrated group. There was a decrease in the intradialytic hypotension events (a mean of 0.68 events vs 1.18) in the overhydrated group after attaining targeted dry weight, however, this was not statistically significant. There was a decrease in the incidence of intradialytic hypertension in the overhydrated group (a mean of 1.82 events vs 2.37). The decrease in the number of intradialytic hypertension events after attaining BCM-targeted dry weight was statistically significant (p-value - 0.017).

**Table 2:** Effect on variables before and after attaining dry weight in the overhydrated group SD - standard deviation, IQR - Interquartile range

S. No	Variables	Expression	Before attaining dry weight	After attaining dry weight	Difference	P-value
1.	Systolic blood pressure (mmHg)	Mean (SD)	147.5(23.3)	141.8(41.3)	5.7	0.943
2.	Diastolic blood pressure (mmHg)	Mean (SD)	81.8(12.2)	78.9(18.5)	2.9	0.368
3.	Urine output (m L)	Median (IQR)	200(10-450)	100(45-325)	100	0.323
4.	Number of antihypertensive medications	Median (IQR)	2.5(2-3)	2(2-3)	0.5	0.125
5.	Intradialytic hypertension (events/session)	Mean (SD)	2.4(3.17)	1.8(3)	0.6	0.017
6.	Intradialytic hypotension (events/session)	Mean (SD)	1.2(2.1)	0.7(1.3)	0.5	0.072

## Discussion

This study aimed at finding out the prevalence of overhydration in patients with CKD undergoing maintenance dialysis using a BCM. All patients enrolled in the study were undergoing maintenance haemodialysis for at least three months. This three-month run-in period was considered as those initiated on haemodialysis were more prone to cardiovascular mortality when sudden changes in ultrafiltration were made.

All the patients enrolled in the study were dialysed with the volume of ultrafiltrate removal based on the clinically observed dry weight. Twenty out of the 30 patients in the overhydrated group had clinical features of volume overload in the form of either pedal edema, pleural effusion or pulmonary edema. Ten of the 30 patients who were found to have overhydration by a BCM were clinically euvolemic. Hence, ultrafiltrate removal based on clinically calculated dry weight may not be accurate and would result in decreased ultrafiltrate removal resulting in persistent overhydration which is associated with high mortality.

The prevalence of overhydration in our study was 27.2%. Wizemann et al. found a prevalence of 21.5% in a study conducted on 269 patients in three centres across Europe. Post HD BCM values were taken to classify patients as overhydrated or non-overhydrated [13]. Onofriescu et al. found the prevalence of overhydration was 26.7% in their study population [2]. Accurate identification of hydration status and fluid management by bioimpedance confers mortality benefits. Onofriescu et al. in a study comparing fluid management using bioimpedance and clinical methods showed a decrease in mortality, relative fluid overload and blood pressure in the bioimpedance group [14]. Vega et al. also showed that any degree of overhydration was associated with higher mortality [15]. Vega et al. found the prevalence of 85% overhydration in patients on haemodialysis with hypertension, however, the prevalence was 23% when overhydration was defined by a cut-off of OH/ECW >15% and 48% when a cut-off of 10%was used [16]. Though BIS was the means used for assessing dry weight, the assessment was done pre-haemodialysis in the study. Ideally, the BCM value calculated post-dialysis was the best method to assess how much excess fluid was present despite apparent adequate removal.

In our study, there was a decrease in mean SBP and DBP by 5mmHg and 3mmHg, respectively. Machek et al. found a reduction of SBP by 25 mmHg and a 35% reduction in antihypertensive medication use [17]. The majority of patients in the study by Machek et al. had an extreme fluid overload which could have resulted in a greater decrease in mean blood pressure. Our study included patients with varied overhydration statuses, with the overhydration index ranging from 1.2 to 6.4, many being clinically euvolemic but BCM showed minimal overhydration. This could explain the lower blood pressure reduction in the study. Hur et al. showed a significant decrease in SBP, left atrial volume index and left ventricular internal dimension in the overhydrated group [18]. However, the study did not follow an objective protocol for the clinical estimation and adjustment of dry weight. Further, the number of patients with absolute overhydration as per OH/ECW ratio in BCM was too low to allow a sufficient estimate of the difference in these parameters in the pre-post dry weight adjustment measures. Onofrescu et al. showed a decrease in mean SBP, but the absolute difference was only 6.5mmHg [14]. Hence, in patients with varied hydration status and a significant proportion of patients having overhydration, the absolute reductions achievable in blood pressure values may be low.

Intradialytic hypertension is a predictor of cardiovascular risk and a marker of mortality in patients on maintenance haemodialysis [12]. Our study showed a significant decrease in the number of intradialytic hypertension events in the overhydrated group, The postulated mechanisms for intradialytic hypertension are sympathetic reactivity, increased renin-angiotensin activation, endothelial dysfunction, vascular stiffness and increased evidence of left ventricular hypertrophy. These changes are mediated by an increase in intravascular volume due to overhydrated state. Hence, as the intravascular volume was corrected there was a significant decrease in the number of hypertensive events in our study.

In this study in spite of ultrafiltrate removal over the interdialytic weight gain, there was a decrease in the number of intradialytic hypotension events, though not clinically significant. The reasons attributable to the decrease in hypotension events could be that once the intravascular volume got corrected, the ultrafiltrate removal rate would have also decreased. Further, as the intravascular volume decreased the preload would decrease resulting in improved cardiac function and thereby a decrease in hypotensive events [19,20].

The study also brought out the difficulties in the attainment of dry weight. Out of 30 patients in the overhydrated group, most patients reached dry weight over a mean duration of 20 weeks rather than the 12 weeks as planned. One of the patients remained overhydrated for a period of 104 weeks. There was a tendency for increased interdialytic weight gain with increased ultrafiltrate removal early in the study; hence, the subsequent volume of ultrafiltrate removed was restricted to prevent intradialytic complications.

Limitations

Adherence to dietary restrictions regarding salt and fluid intake could not be strictly implemented. Left ventricle function which has a bearing on intradialytic events was not assessed for all the patients in the overhydrated group.

## Conclusions

The study validates the use of BCM in our study population. The study shows a significant decrease in intradialytic hypertension and a non-significant decrease in the mean SBP and DBP, intradialytic hypotension and a decrease in the number of antihypertensive medications for blood pressure control. The decrease in intradialytic hypertension events was due to change in intravascular volume through BCM-adjusted dry weight attainment. Further, large randomized controlled studies are needed to identify the benefits of BCM over clinical methods in target dry weight attainment and their effect on intradialytic complications and control of blood pressure.
